# Intraductal cryobiopsy via percutaneous cholangioscopy for biliary strictures: a multicenter feasibility study

**DOI:** 10.1055/a-2728-8013

**Published:** 2025-12-19

**Authors:** Jan Peveling-Oberhag, Christian Gerges, Jörg Albert, Lukas Welsch, Philip Grunert, Gilbert Rahe, Alexander Dechene, Axel Eickhoff, Matthias Dettmer, Walter Linzenbold, Markus Enderle, Thomas Rösch, Katharina Zimmermann-Fraedrich

**Affiliations:** 1 14881 Gastroenterology, Gastrointestinal Oncology, Hepatology and Infectious Diseases, Klinikum Stuttgart, Stuttgart, Germany; 2 Clinic for Gastroenterology, Hepatology and Transplant Medicine, University Hospital Essen, Essen, Germany; 3 Internal Medicine II, Gastroenterology, Hepatology, Interventional Endoscopy, Infectiology, Health and Medical University, Campus Helios Clinic Krefeld, Krefeld, Germany; 4 39790 Internal Medicine II, Clinic for Gastroenterology, Diabetology and Infectious Diseases, Klinikum Hanau GmbH, Hanau, Germany; 5 9211 Internal Medicine 6, Gastroenterology, Klinikum Nürnberg, Nürnberg, Germany; 6 39790 Klinik für Gastroenterologie, Klinikum Hanau gGmbH, Hanau, Germany; 7 14881 Institute for Pathology, Klinikum Stuttgart, Stuttgart, Germany; 8 27210 Institute of Tissue Medicine and Pathology, University of Bern, Bern, Switzerland; 9 27657 Research Department, Erbe Elektromedizin GmbH, Tübingen, Germany; 10 Interdisciplinary Endoscopy, Universitätsklinikum Hamburg-Eppendorf, Hamburg, Germany

## Abstract

**Background:**

Tissue diagnosis of biliary strictures is challenging and often requires multiple methods. Cryobiopsy, which is well established in bronchoscopy with high tissue yield, is presented here for the first time as a proof-of-principle feasibility study performed via the percutaneous route for biliary strictures.

**Methods:**

Patients undergoing percutaneous cholangioscopy for intraductal diagnosis of biliary strictures underwent six forceps biopsies and three cryobiopsies in a randomized order. The main objective was to assess feasibility, defined as the retrieval of at least one adequate sample per method per patient.

**Results:**

Among 15 patients (53% women; mean age 60.2 years), all had at least one adequate sample obtained by each method. Cryobiopsy yielded significantly larger (8.54 vs. 1.87 mm ^2^ ; *P* < 0.001) and more representative specimens (97.6% vs. 74.7%; *P* = 0.001). It also scored higher on overall histologic quality on a Likert scale of 0–6 (5 vs. 4; *P* < 0.001) and had more artifact-free areas (93.5% vs. 85.5%; *P* = 0.01). No bleeding or perforations occurred; only minor adverse events were reported and these resolved with standard treatment.

**Conclusions:**

This feasibility study showed that intraductal cryobiopsy via percutaneous cholangioscopy yielded larger samples and may enable more detailed histologic assessment than forceps biopsies. Further studies will evaluate its accuracy, safety, and potential for use with peroral cholangioscopy during endoscopic retrograde cholangiopancreatography.

## Introduction

Endoscopic forceps biopsy has long been the standard in luminal endoscopy, with high accuracy for epithelial lesions, but tissue diagnosis during endoscopic retrograde cholangiopancreatography (ERCP) remains less accurate and less well standardized 1 2 . Advanced imaging techniques such as confocal microscopy 3 and cholangioscopic visual assessment using artificial intelligence 4 , as well as endoscopic ultrasound (EUS)-guided sampling 5 , have been added to the spectrum. Multiple methods are often combined to reach a definitive diagnosis 6 .

Diagnostic accuracy for biliary strictures remains variable, particularly for detecting or ruling out malignancy, which carries major clinical implications. Most available data come from retrospective case series – some comparative, few prospective or randomized – with variable results across techniques 7 8 . A 2015 meta-analysis 9 and a 2023 ASGE survey 1 reported sensitivities of 40%–60% for intraductal biopsy and brushing, while cholangioscopic biopsies showed slightly better performance (70%–75%). Given the demands of personalized cancer therapy and the need for detailed histologic analysis, a more reliable intraductal biopsy method is needed, ideally matching the effectiveness of standard luminal biopsies.

Cryobiopsy is well established for tissue sampling in bronchoscopy 10 11 12 , and a similar probe has shown promising results in the biliary system in ex vivo studies 13 and a single patient case 14 . We now report the first multicenter pilot series comparing cryobiopsy with percutaneous cholangioscopic forceps biopsy. The cryoprobe, similar to its bronchoscopic counterpart, is evaluated here as a proof of principle before its potential development for ERCP use. Because of its limited length, only percutaneous application was feasible.

## Methods

### Patients and procedures

Patients undergoing percutaneous cholangioscopy with tissue sampling for biliary strictures were enrolled at five centers between March (first treatment) and August 2024 (last visit). Institutional Review Board approval was obtained at all sites. Patients were included if they had a biliary stricture without a mass lesion or clear diagnosis based on their history or a prior biopsy, and a tissue diagnosis was still required for further management. This also included patients with primary sclerosing cholangitis (PSC) presenting with a dominant stricture, where access via ERCP or enteroscopy was not feasible. Percutaneous drainage was established prior to cholangioscopy as necessary. Indications, plus patient and procedural data, including cryo activation time, number of biopsy attempts, gas pressure, procedure duration, and biopsy area, were recorded.

Cholangioscopy was performed using a single-use cholangioscope (SpyGlass Discover; Boston Scientific, USA) that was introduced via the percutaneous tract according to each center’s standard procedure, typically using a sheath and/or a guidewire. The cholangioscopic visual appearance was used to guide biopsies. The biopsy techniques were applied in randomized order (sealed envelopes), with either six forceps biopsies or three cryobiopsies performed first. Each sample was placed in a separate pathology vial (nine per patient). A post-biopsy drain was routinely inserted, as per the standard practice at all participating centers.

#### Forceps biopsy

SpyBite Max biopsy forceps (Boston Scientific) were used for all forceps biopsies. Any additional biopsies taken beyond the six study samples were excluded from the analysis and were used solely for clinical purposes.

#### Cryobiopsy

The cryo principle is demonstrated using a water container, where the frozen attachment is visible. Percutaneous cholangioscopy is then performed in a patient with intrabiliary projections to diagnose or rule out cancer. The probe is introduced and pressed against the lesion; after the foot pedal has been pressed for 4–5 seconds, the probe and cholangioscope are removed together, with a large attached specimen clearly visible.Video 1

**Fig. 1:**
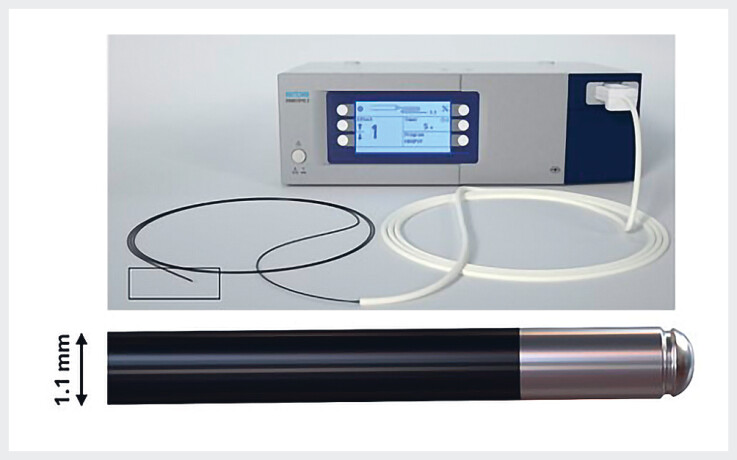
The ERBECRYO 2 device with its corresponding single-use 1.1-mm cryoprobe.

The cryobiopsy technique is similar to that used in bronchoscopy 10 ; the equipment used is described in **Table 1s** , see online-only Supplementary material. A 1.1-mm flexible cryoprobe ( Fig. 1 ) was connected to the ERBECRYO 2 unit, which automatically recognized the probe and selected the appropriate settings. Functionality was tested in sterile water with a 5-second activation via foot pedal. For biopsy, the probe was positioned tangentially or perpendicularly to the target lesion and activated for 4–5 seconds, guided by an acoustic signal. It was then withdrawn, together with the cholangioscope, through the percutaneous tract, remaining activated until the specimen was released into fluid and then formalin. This process was repeated twice, for a total of three attempts, even if no tissue was obtained ( Video 1 ).

#### Histopathology

Biopsies were evaluated by a specialized gastrointestinal (GI) histopathologist at each center, who was blinded to the acquisition method. For intraindividual comparison, the same standard histopathologic criteria were applied to both biopsy types from each patient.

#### Follow-up

Follow-up was conducted before discharge and on day 30 ± 7 via telephone. Patients were asked about any symptoms potentially related to the procedure (e.g. bleeding or pain), as well as any medical consultations or hospital admissions. To ensure the diagnosis of benign biliary stricture, follow-up was subsequently conducted clinically and by imaging for at least 6 months.

### Outcomes and definitions

#### Main objective

The main objective was the feasibility of percutaneous cryobiopsy in the bile duct, meaning the successful retrieval of at least one cryobiopsy specimen per patient.

#### Further objectives

- **Success rate** Percentage of successful biopsy retrievals per technique, based on total attempts (45 cryobiopsies and 90 forceps biopsies).
- **Biopsy volume** Estimated by the overall mean area (mm ^2^ ) per biopsy, calculated by summing all section areas per biopsy and patient (2D surrogate for 3D volume).
- **Biopsy quality** Assessed using a histopathologic Likert scale for each technique (adapted from Wirsing et al. 13 ).
- **Representativeness** Defined as a score >2, indicating moderate limitations but sufficient quality to assess relevant morphologic and histologic features.
- **Crush artifacts** Graded by the percentage of artifact area per sample: none; low, 1%–19%; moderate, 20%–39%; or high, >40%.
- **Procedure time** Measured overall and separately for each biopsy method.
- **Adverse events** Adverse events included death, bleeding, post-procedural bleeding, perforation, infection, and abscess formation. Events were categorized according to ISO 14155:2020 and MDR 2017/745 (Articles 2 and 80), and graded by severity (mild, moderate, severe).

### Statistics

For descriptive statistics, categorical variables are presented as counts and percentages, and continuous variables as mean (SD) or median with interquartile range (IQR; 25th–75th percentile). Normally and non-normally distributed data were compared using Student’s *t* test and Mann–Whitney *U* test, respectively. Differences in means or medians are reported with 95%CIs. A two-sided *P* value <0.05 was considered statistically significant. Analyses were performed using GraphPad Prism, version 9.5.1 (GraphPad Software LLC).

As this was the first in vivo feasibility study, no formal power or sample size calculation was performed. A sample of 15 patients was chosen to assess the feasibility of percutaneous bile duct biopsy and inform future large randomized multicenter trials.

### Safety assessment and reporting requirements

Participant safety was monitored throughout the study. The investigation was approved by the Ethics Committee of the University of Tübingen (No. 401/2023MP1), was performed according to the Helsinki declaration, and was reported to the German Federal Institute for Drugs and Medical Devices (BfArM; EUDAMED No. CIV-23–06–043299), in accordance with the Medical Device Law Implementation Act (MPDG) and EU MDR 2017/745.

## Results

A total of 15 patients were included (7 men; mean age 60.2 [SD 18.5]; body mass index 23.1 kg/m ^2^ ) ( Table 1 ). The five centers enrolled seven, four, two, one, and one patients each. The mean (SD) total procedure time was 13.58 (7.28) minutes, defined from cholangioscope insertion to the final biopsy. The mean (SD) times were 7.14 (2.40) minutes for forceps biopsy and 6.45 (6.45) minutes for cryobiopsy ( *P* = 0.74).

**Table:** **Table 1** Demographic and procedural data for the 15 patients with undiagnosed biliary stricture who underwent biopsies using forceps and cryobiopsy.

	All	Forceps biopsy	Cryobiopsy	95%CI for mean difference	*P* value
Age, years	60.2 (18.5)				
Sex, female, n	8				
Procedural time, mean (SD), minutes ^1^	13.6 (7.3)	7.1 (2.4)	6.5 (4.6)	−2:3 to 3:3	0.74
Histologic results					
Adequate samples, %					
Per patient		100 (15/15)	100 (15/15)		>0.99
Per biopsy attempt ^1^		96 (87/90)	93 (42/45)		0.40
Representative specimens obtained, % ^2^		74.70 (65/87)	97.60 (41/42)		0.001
Area, mean (SD), mm ^2 2^		1.87 (1.86)	8.54 (6.00)	−8.07 to −5.27	<0.001
Crush artifacts, median (IQR) ^3^		1 (1)	1 (1)	0 to 1	0.06
Artifact-free areas, mean (SD), %		85.5 (19.3)	93.5 (7.0)	−14.1 to −1.9	0.01
General assessment score, median (IQR) ^4^		4 (3)	5 (2)	−2 to 0	<0.001
^1^ Six biopsies were taken by forceps (total of 90) and three by cryobiopsy (total of 45). ^2^ For details of the determination of the specimen being representative (yes/no) and for area calculation as a surrogate parameter for volume see Methods. ^3^ Graded 0 (none) to 3 (high). ^4^ Likert scale, 0–6.					

**Fig. 2:**
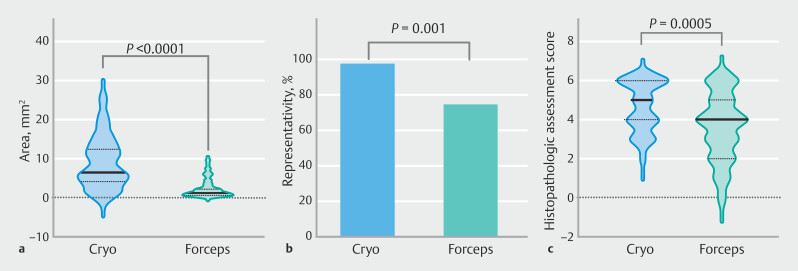
Graphical comparison of cryobiopsy and standard forceps biopsy with cryobiopsy resulting in: **a** significantly larger biopsies; **b** more representative specimens; **c** specimens with a higher histopathologic assessment score.

**Fig. 3:**
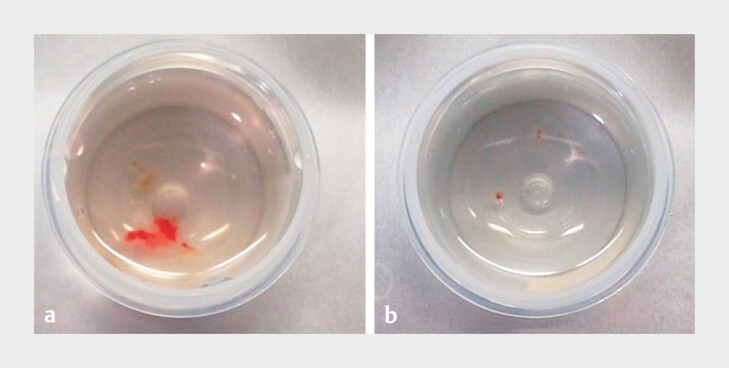
Example macroscopic specimens of biliary tissue obtained using: **a** the cryoprobe; **b** forceps biopsy via percutaneous cholangioscopy.

**Fig. 4:**
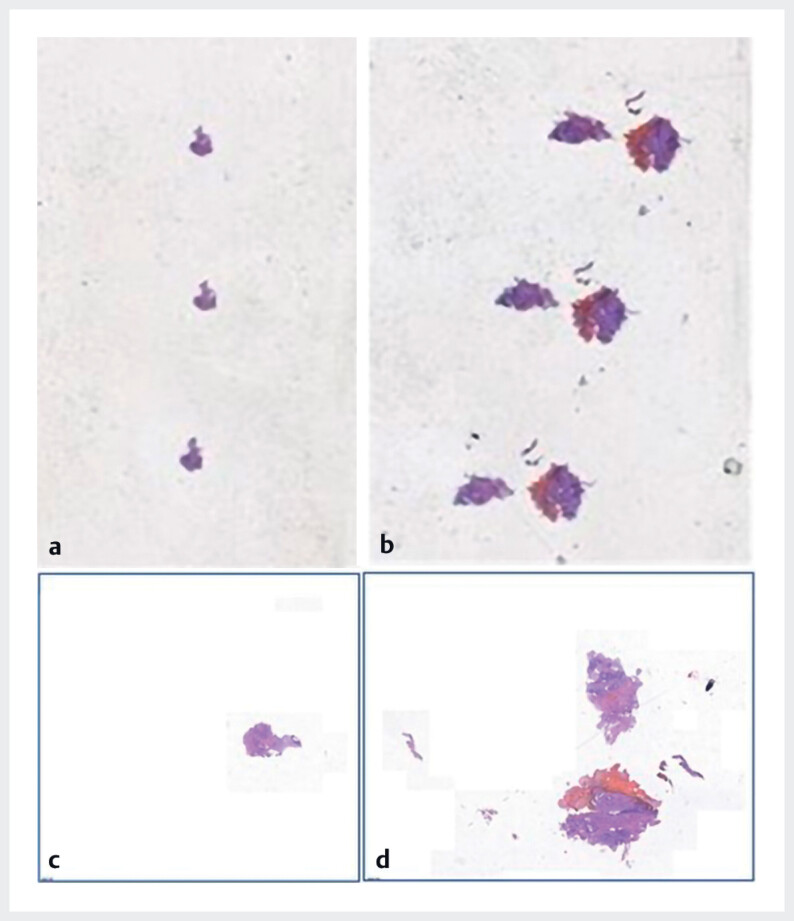
Histologic appearance of the obtained tissue: **a,b** displayed on the slides; **c,d** at 1.5× magnification; **a,c** forceps biopsy; **b,d** biopsy using the cryoprobe.

Histology results and final diagnoses are summarized in Table 1 and Fig. 2 . At least one adequate sample was obtained in all patients and by both biopsy techniques. Adequate specimens were retrieved in 42/45 (93.3%) cryobiopsies and 87/90 (96.7%) forceps biopsies. Cryobiopsies yielded significantly larger (8.54 [SD 6.00] vs. 1.87 [SD 1.86] mm ^2^ ; *P* < 0.001) and more representative samples (97.6% vs. 74.7%; *P* = 0.001), with a higher percentage of artifact-free areas (93.5% [SD 7.0%] vs. 85.5% [SD 19.3%]; *P* = 0.01) and higher histology scores on the 6-point Likert scale (5 [IQR 2] vs. 4 [IQR 3]; *P* < 0.001). Notably, the first cryobiopsy was representative in 100% of cases vs. 57.1% for the first forceps biopsy. Representative images are shown in Fig. 3 and Fig. 4 ; clinical and histologic data are detailed in Table 2 . There was no significant difference in biopsy size based on the sequence of the biopsy technique. Neither cryobiopsy (first, 7.5 [SD 4.7] mm ^2^ ; second, 9.3 [SD 9.3] mm ^2^ ; *P* = 0.34) nor forceps biopsy (first, 1.6 [SD1.8] mm ^2^ ; second, 2.2 [SD 2.0] mm ^2^ ; *P* = 0.17) showed statistically significant differences ( **Fig. 1s** ).

**Table:** **Table 2** Individual patient data.

Age, years; sex	Diagnostic setting	Final diagnosis	Diagnosis by:		Follow-up, months	Outcome
			Forceps	Cryo		
81; male	BDA stenosis after pancreatectomy (high grade IPMN)	Inflammatory stenosis	X	X	3	Stricture resolution
59; female	Late BDA stenosis after LTX ^1^	Inflammatory stenosis	X	X	12	Switched to ERCP
28; female	Late LHD stenosis after LTX (cryptogenic liver failure) ^1^	Inflammatory stenosis	X	X	11	Stricture resolution
61; female	BDA stenosis after extended right hemihepatectomy (CCA)	Recurrence of CCA	X	X	4	Cancer death
73; female	BDA stenosis after right hemihepatectomy (PSC)	Inflammatory stenosis	X	X	3	Death from liver cirrhosis
19; female	Late stenosis of left HD after LTX ^1^	Inflammatory stenosis	X	X	8	Switched to ERCP
59; male	BDA stenosis after left hemihepatectomy (LGIN)	Inflammatory stenosis	X	X	6	Stricture resolution
44; male	Late BDA stenosis in PSC	Inflammatory stenosis	X	X	11	Repeated PTBDE
77; male	Late biliary stenosis after LTX for HCC	Exclusion of malignancy	X	X	11	Repeated ERCP
76; female	BDA stenosis after pancreatectomy for cancer	Chronic ulcer ^2^	X	X	8	Repeated PTBDE
67; female	BDA stenosis after surgery for CCA	Local recurrence	0	0	3	PTBDE, fatal ulcer bleeding
64; male	LHD stenosis after right hepatectomy for CRC metastases	Local recurrence	0	X	8	Palliative chemotherapy
77; male	Suspicious hilar stricture	CCA	X	X	3	Cancer death
45; female	Suspicious distal CBD stricture	Pancreatic carcinoma	0	0	–	Pancreatectomy, T1N1
73; male	BDA stricture after surgery for lymphoma	Inflammatory stenosis	X	X	7	Lymphoma, stable
BDA, biliodigestive anastomosis; CBD, common bile duct; CCA, cholangiocarcinoma; CRC, colorectal carcinoma; ERCP, endoscopic retrograde cholangiopancreatography; HCC, hepatocellular carcinoma; IPMN, intraductal papillary mucinous neoplasm; LGIN, low grade intraepithelial neoplasia; LHD, left hepatic duct; LTX, liver transplant; PSC, primary sclerosing cholangitis; PTBDE, percutaneous transhepatic biliary drainage exchange. ^1^ Biopsy was indicated to exclude neoplasia in late strictures after liver transplantation (LTX) for primarily benign disease under immunosuppression. ^2^ Imaging and biopsy negative, further follow-up pending.						

All patients were assessed for adverse events at discharge and at 30 days. Three reported post-procedural abdominal pain, two had fever, one developed cholangitis, and one had a wound infection at the access site. None of the events were directly linked to either of the biopsy techniques. No bleeding or perforation occurred. All adverse events resolved with analgesics and/or antibiotics.

## Discussion

Tissue diagnosis can be difficult in the biliary tract; even forceps biopsy under direct vision with cholangioscopy may often not provide sufficient samples, in part due to tissue hardness and the angle of biopsy. These factors help explain the well-known limitations in obtaining sufficient tissue samples from the bile duct, where specimens are often much smaller than the actual cup size of the forceps. This limitation was one of the driving forces behind the development of cryobiopsy, which relies on tissue freezing, adherence, and traction to retrieve samples. We present the results of a pilot, proof-of-principle study on the use of biliary cryobiopsy for biliary strictures. The findings are promising in terms of the technical feasibility and, notably, the superior quality of the histologic specimens. Cryobiopsy yielded significantly larger and more representative samples than standard forceps biopsy, with fewer artifacts and higher histopathologic assessment scores.

To align with current clinical practice, twice as many forceps biopsies were performed; however, cryobiopsy may ultimately achieve high diagnostic yields with fewer samples. This could prove valuable for more detailed histologic analysis or when limited sampling is preferred, particularly once ERCP-compatible cryoprobes are developed. For this study, we used a short cryoprobe adapted from bronchoscopy to assess the initial feasibility of the biliary cryobiopsy technique in general, and employed a randomized design to ensure comparability with forceps biopsy.

Because of the limited sample size, our study could not assess diagnostic accuracy or provide a comprehensive safety analysis. As both biopsy methods, forceps and cryoprobe, were used in the same patients, attribution of any complications to a specific technique is currently limited. No severe procedure-related complications (e.g. bleeding or perforation) were observed. Safety will need further evaluation, particularly in the ERCP setting.

Following this feasibility study on tissue yield, it is clear that further prospective and comparative studies with adequate outcome parameters are needed to define the clinical role of cryobiopsy in biliary strictures. Such future cryobiopsy studies should consider pretest probability based on clinical context, such as patient age or underlying conditions like PSC or secondary sclerosing cholangitis, as diagnostic needs differ across these groups. This has often been overlooked, contributing to variable accuracy and limited data on clinical impact.

Historically, the sensitivity of brush cytology and forceps biopsy under fluoroscopy has ranged between 40% and 60%, as reported in reviews from 2015 9 and 2023 1 . With the wider use of cholangioscopy and direct biopsy, diagnostic yield has improved. The 2023 ASGE survey reported a 27% incremental gain across four comparative studies 1 . The role of artificial intelligence in enhancing cholangioscopic imaging, alone or combined with clinical and laboratory data, remains an area for future exploration 15 16 . There may be a correlation between tissue volume and diagnostic yield or accuracy, but limitations are especially prominent in biliary malignancies with a high proportion of inflammatory and fibrous components. Our results may be encouraging in that respect, and further studies are warranted.

Currently, biliary tissue acquisition often involves multiple diagnostic methods to achieve adequate clinical accuracy. With the rise of personalized medicine and advances in chemo- and immunotherapies in both palliative and neoadjuvant settings, the demand for detailed histologic analyses, including immunohistochemistry and tumor sequencing, has grown 17 18 . In many cases, a simple malignancy diagnosis is no longer sufficient; more tissue is needed for precise treatment planning. Biliary cryobiopsy, especially with future ERCP-compatible probes, may offer an advantage by providing larger, higher quality samples to meet these evolving clinical needs. This should be further investigated using a longer probe compatible with ERCP in larger prospective multicenter trials to generate meaningful data on clinical utility.

## Supplementary Material

Supplementary Material Zusatzmaterial
