# Supplementary material for: Intraductal cryobiopsy via percutaneous cholangioscopy for biliary strictures: a multicenter feasibility study
Source: Endoscopy. 2025 Dec 19;58(4):409–15. doi: 10.1055/a-2728-8013 (PMC13004657; doi:10.1055/a-2728-8013)

Thieme

SUPPLEMENTARY MATERIAL

Intraductal cryobiopsy via percutaneous cholangioscopy for biliary strictures: a multicenter feasibility study

Jan Peveling-Oberhag*, Christian Gerges*, Jörg Albert, Lukas Welsch, Philip Grunert, Gilbert Rahe, Alexander Dechene, Axel Eickhoff, Matthias S. Dettmer, Walter Linzenbold, Markus Enderle, Thomas Rösch, and Katharina Zimmermann-Fraedrich

* Joint first authors.

Table 1s: Equipment used for biliary cryobiopsies

	Technical Specification	Value
Flexible cryoprobe	Single Use	Yes
	Feature	Flexible
	Distal length	1150 mm
	Diameter	1.1 mm
Erbecryo 2 Device	Effect Level	1
	Cooling Agent	CO_2
	Pressure	45 – 65 bar
Accesso-ries	Footpedal	
	CO2 tank	10 kg
	Device Cart	

Figure 1s Biopsy area achieved by cryobiopsies and forceps biopsies depending on order.

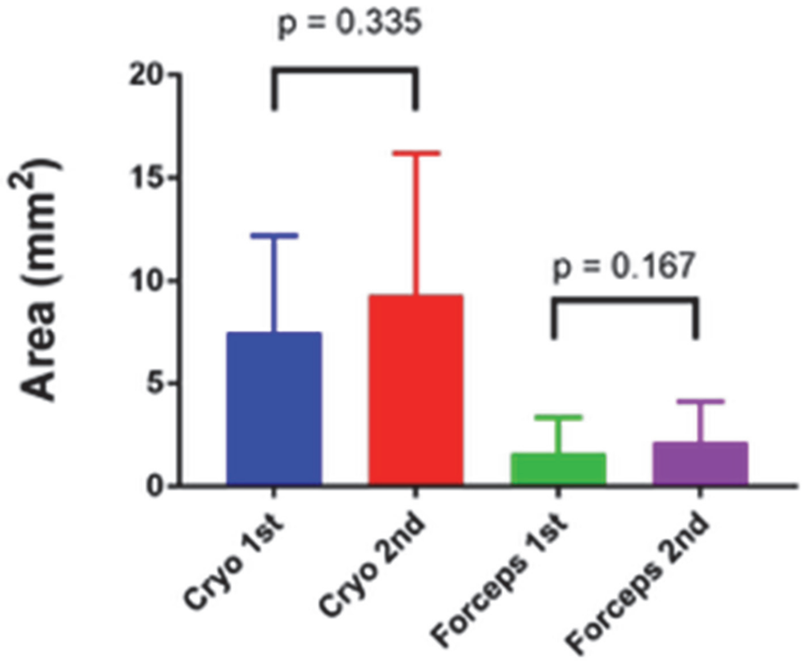

Supplement: Supplementary file 2 — Supplementary Material [file 10-1055-a-2728-8013_27498811.pdf]
